# Functions and mechanisms of RNA m^6^A regulators in breast cancer (Review)

**DOI:** 10.3892/ijo.2024.5674

**Published:** 2024-07-26

**Authors:** Yibei Yang, Feng Gao, Lanqi Ren, Ning Ren, Junjie Pan, Qiaoping Xu

**Affiliations:** 1 Department of Clinical Pharmacology, Key Laboratory of Clinical Cancer Pharmacology and Toxicology Research of Zhejiang Province, Affiliated Hangzhou First People's Hospital, Cancer Center, Westlake University School of Medicine, Hangzhou, Zhejiang 310006, P.R. China; 2 Fourth Clinical Medical College of Zhejiang Chinese Medical University, Hangzhou, Zhejiang 310051, P.R. China; 3 Department of Urology, Hangzhou Hospital of Traditional Chinese Medicine, Hangzhou, Zhejiang 310000, P.R. China

**Keywords:** breast cancer, N^6^-methyladenosine, m^6^A modification regulator, mechanistic pathways, RNA

## Abstract

Breast cancer (BC) is a major malignant tumor in females and the incidence rate of BC has increased worldwide in recent years. N^6^-methyladenosine (m^6^A) is a methylation modification that occurs extensively in eukaryotic RNA. The abnormal expression of m^6^A and related regulatory proteins can activate or inhibit certain signal pathways or oncogenes, thus affecting the proliferation, metastasis and prognosis of BC. Numerous studies have shown that m^6^A regulator disorder exists in BC, and this disorder can be reversed. Therefore, m^6^A is predicted as a potential therapeutic target for BC. However, the molecular mechanism of m^6^A RNA methylation regulating the occurrence and development of BC has not been comprehensively elucidated. In this review article, the functions of various m^6^A regulators and the specific mechanisms of certain regulators of the progress of BC were summarized. Furthermore, the dual role of RNA methylation in tumor progression was discussed, concluding that RNA methylation can not only lead to tumorigenesis but at times give rise to inhibition of tumor formation. In addition, further comprehensive analysis on mechanisms of m^6^A regulators in BC is conducive to screening effective potential targets and formulating targeted treatment strategies, which will provide new methods for the prevention and treatment of BC.

## 1. Introduction

Breast cancer (BC) is a common malignant tumor and its incidence rate has shown an overall upward trend in the past decade (1). Despite the progress in both understanding and treating BC, nearly 30% of patients suffer from recurrence or metastasis due to the deficiency of effective treatment or prevention strategies, which is the main reason for BC-related mortality (2). The extensively used classification for BC comprises Luminal A, Luminal B, human epidermal growth factor receptor (EGFR) 2 (HER2) overexpression and triple-negative BC (TNBC) (3). Studies have indicated that with early diagnosis and timely treatment, the overall survival of nonmetastatic BC and *de novo* metastatic BC (MBC) has been evidently improved. However, for recurrent MBC and elderly patients, there has been no improvement in decades (4). Hence, investigating the molecular mechanisms underlying the onset and progression of BC, and enhancing the capacity for monitoring BC treatment efficacy or identifying promising therapeutic targets, are of immense importance for precise diagnosis, efficient stratified management and the development of more refined treatment strategies for BC.

N^6^-methyladenosine (m^6^A) is the most prevalent internal mRNA modification in eukaryotes, which is installed by the methyltransferase complex (MTC) and removed by demethylases (5). It has been established as a widespread regulatory mechanism that controls gene expression in diverse physiological and pathological processes, including cancer (6,7). As the most universal epigenetic RNA modification, m^6^A plays a crucial role in regulating RNA stability, decay, splicing, transport and translation, thus affecting tumor progression significantly (8,9).

Abnormal m^6^A regulators have been recognized as new anticancer drug targets according to the close correlation between aberrant m^6^A modification and the occurrence, progression and prognosis of tumors (10). As the m^6^A modification and its associated factors are significantly dysregulated in cancers, gaining a comprehensive understanding of their roles in tumorigenesis and cancer progression will provide in-depth insight into the development of new therapeutic strategies for cancer treatment. The present review aims to summarize the current understanding of m^6^A modification and its functions in biological processes and cancers, with a particular focus on its mechanisms and roles in BC progression. Furthermore, the functions of m^6^A in DNA damage response, genomic instability and metabolic reprogramming were summarized.

## 2. BC

BC originates from mammary duct epithelial cells, which is the malignant tumor type with the highest incidence and mortality rates for women worldwide, accounting for ~30% of cancers in females (11-13). Clinical manifestations of BC may include breast lumps, nipple discharge and breast skin changes. However, early symptoms of part of breast cancer are not obvious or characteristic, which increases the difficulty of early identification.

BC exhibits apparent heterogeneity. According to the status of hormone receptors [estrogen receptor (ER) and progesterone receptor (PR)] and HER2, BC can be classified into three primary subtypes: Luminal ER-positive and PR-positive, which can be further categorized as luminal A and B, HER2-positive BC and TNBC (14,15). This BC classification based on biomarkers provides a foundation for further research and more precise determination of prognosis and selection of personalized treatments (13). For instance, the systemic treatment of nonmetastatic BC typically varies based on the subtype: Hormone receptor-positive tumors generally exhibit improved outcomes with endocrine therapy, while erb-b2 receptor tyrosine kinase 2 (ERBB2)-positive tumors typically require ERBB2-targeted antibodies or small-molecule inhibitors in combination with chemotherapy. By contrast, patients with triple-negative tumors tend to display greater sensitivity to chemotherapy (16).

The prognosis for BC varies among the different subtypes, which may be the most significant factor. Luminal A is the molecular subtype with the highest proportion in BC, exhibiting the lowest malignancy and the highest 5-year survival rate. The prognosis of patients with luminal B is slightly worse than that of patients with luminal A according to statistics (17). Compared to other subtypes, patients who are HER2-positive and those with TNBC often exhibit greater invasiveness, higher potential for recurrence and metastasis, and a poorer prognosis. It poses a significant challenge in the treatment of BC (18-20). In addition, other factors such as advanced age at diagnosis, later stage of cancer progression, metastasis, genetic predisposition and even high parity may also contribute to a worse prognosis (17).

A large portion of BC cases can be attributed to reproductive and hormonal factors (early menarche, late menopause, later primiparity age) (21), as well as lifestyle factors (e.g. overweight, lack of exercise, alcohol intake and smoking) (22). It has been proved that long-term contraceptives or menopausal hormone therapy with a combination of estrogen and progesterone raise the risk of BC as well (23). Familial inheritance is another universally acknowledged formidable hazard factor for BC. Women with a family history have a 2-to-4-fold increased probability of suffering from BC compared to others, with younger diagnosis ages and even higher mortality rates (24,25). Germline mutations in genes such as ATM serine/threonine kinase, BRCA1 DNA repair associated (BRCA1), BRCA2, checkpoint kinase 2 and partner and localizer of BRCA2 are frequently associated with an increased risk of developing BC (26,27). However, such mutations are rare in the general population.

## 3. M^6^A

M^6^A refers to methylation modification on the 6th nitrogen atom of RNA adenylate (28), which is the most universal internal messenger RNA modification in eukaryotes. M^6^A methylation can be found in mRNA, micro (mi)RNAs and long non-coding (lnc)RNAs to monitor and regulate their stability, translation, splicing and transport (29,30). The majority of m^6^A-modified mRNAs contain only one m^6^A site, while others contain 20 or more. Sequence analysis indicated that these sites emerge uniformly on the consensus RRACH motif and are not randomly distributed throughout the entire transcript. Instead, they tend to be concentrated in the 3'-untranslated region (UTR) near the stop codon (31,32). Furthermore, m^6^A modification is dynamic and reversible, which is installed by methyltransferases ('writers'), recognized by RNA-binding proteins ('readers') and removed by demethylases ('erasers') (28). Methyltransferase complex, an enzyme that catalyzes the methylation of m^6^A, consists of several core proteins. The so-called 'reader' can identify and bind to m^6^A methylated targets to carry out follow-up biological processes (33). The erasers are in charge of removing relevant markers through dynamic regulation, but they exert activity only in specific tissues or under certain disease-relevant conditions. These proteases constitute a vital regulator system required for different stages of gene expression that affects specific biological processes (34). However, when these regulators are dysregulated, particularly in tumors, they may stimulate the occurrence of tumors, proliferation and metastasis of cancer cells (9,35). Hence, investigating disorders in m^6^A levels could be immensely significant for detecting cancer and monitoring its treatment.

### m^6^A writers

m^6^A methyltransferase complex, which is named 'writers' as well, is composed of core proteins methyltransferase-like 3 (METTL3), METTL14, Wilms tumor 1-associated protein (WTAP) and other proteins. METTL3 and METTL14 form stable heterodimers, which are responsible for the majority of m^6^A sites in mRNA (34). Among them, METTL3 functions as the fundamental catalytic enzyme by binding to S-adenosylmethionine as a subunit, and is capable of enhancing the translation of most oncogenic mRNAs (31,36). METTL14 acts as an allosteric activator of METTL3, thereby stabilizing its structure and recognizing target RNAs (37). WTAP is essential for the proper localization of the METTL3-METTL14 complex to nuclear speckles and for the enhancement of its catalytic activity (38). Furthermore, m^6^A methylation is regulated by several other proteins. METTL16 has been demonstrated to function independently and regulate m^6^A modification in several RNAs. Specifically, in the nucleus, it deposits N^6^ into specific mRNA targets, while separately stimulating translation in the cytoplasm (39). KIAA1429 [also known as vir-like m^6^A methyltransferase-associated protein (VIRMA)], a newly confirmed ingredient of the m^6^A methyltransferase complex, is crucial in guiding the process of regioselective m^6^A deposition. It means that KIAA1429 can mediate preferential m^6^A deposition in the 3'UTR and around the termination codon (40). RNA binding motif protein 15/15B (RBM15/15B) can attract and bind cone proteins complexes, directing them to specific RNA positions (41). Zinc finger CCCH domain-containing protein 13 (ZC3H13) serves as an anchor for the complex (WTAP, VIRMA and Cbl proto-oncogene like 1) within the nucleus so as to promote m^6^A methylation and mouse embryonic stem cell self-renewal (42). The summarized functions of m^6^A writers are presented in Table I.

### m^6^A erasers

The eraser enzyme is capable of facilitating m^6^A demethylation through the involvement of two proteins, namely Fat mass and obesity-associated protein (FTO) and AlkB homolog 3/5 RNA demethylase (ALKBH3/5) (33). FTO and ALKBH5, which belong to the α-ketoglutarate-dependent dioxygenase family, only work in the presence of oxygen, ferrous ions and α-ketoglutarate (43). They initiate the conversion of m^6^A into N^6^ hydroxymethyl adenosine and subsequently into N^6^ formyl adenosine (f^6^A). Ultimately, f^6^A is hydrolyzed to adenosine to complete the demethylation process (43). FTO is the first m^6^A demethylase and it exhibits a strong correlation with weight gain, obesity and other metabolic diseases in humans (44). FTO is situated in both the nucleus and cytoplasm, and directly participates in the regulation of multiple pre-nuclear mRNA processing pathways, as well as other processes such as mRNA splicing (45). The second eraser, ALKBH5, is located in the nucleus and regulates gene expression mainly by mediating the transportation, metabolism and assembly of mRNA (43). The expression level of FTO and ALKBH5 affects the level of m^6^A in mRNA. Numerous studies have reported that the overexpression of m^6^A erasers is closely related to the occurrence and development of cancer (43,44,46). The functions of m^6^A erasers are summarized in Table II.

### m^6^A readers

The reader is another participant in the dynamic and reversible regulation of m^6^A methylation, which can recognize and bind to m^6^A targets. The readers comprise the YTH domain family of proteins (YTHDC1/2, YTHDF1/2/3), insulin-like growth factor 2 mRNA binding protein (IGF2BP1/2/3), the heterokaryotic nuclear RNA protein family [heterogeneous nuclear ribonucleoprotein C (HNRNPC), HNRNPG] and eukaryotic initiation factor 3 (eIF3) (47). Diverse species rely on different m^6^A readers to perform specific biological functions.

The YTH family members are the most vital readers with a conservative m^6^A binding domain. YTHDF2 recruits RNA decay mechanism factor (C-C motif chemokine receptor 4-NOT deaminase complex) directly, which has an important role in accelerating the degradation of m^6^A-modified RNA (48). Unlike YTHDF2, YTHDF1 may neither take part in mRNA decay directly nor alter the total methylation mRNA level, but it facilitates translation by interacting with translation initiation factors and ribosomes (49,50). YTHDF3 is considered an assistant to boost the translation or degradation of target RNA with two other YTHDF members (51). YTHDC1 facilitates exon inclusion in the nucleus by recruiting splicing factor 3 (SRSF3) and preventing SRSF10 from binding to mRNA (52). It can also promote the nuclear export of m^6^A-labeled mRNA by interacting with nuclear transport receptors, and it is involved in promoting the stability of mRNA transcripts (47,53). YTHDC2 has 3′→5′ RNA helicase activity and improves the translation efficiency of target mRNA (54).

**Figure 1:**
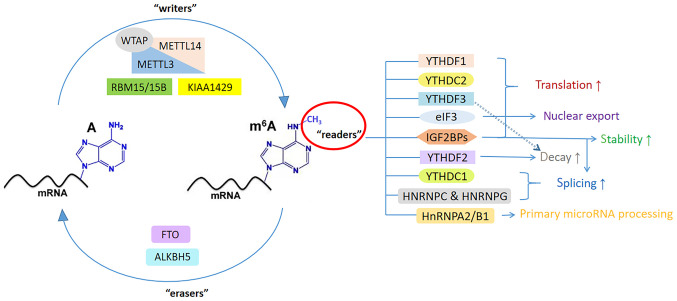
Specific functions of m^6^A writers, erasers and readers. m^6^A, N^6^-methyladenosine; METTL3, methyltransferase-like 3; WTAP, Wilms tumor 1-associated protein; KIAA1429/VIRMA, vir-like m6A methyltransferase-associated protein; RBM15/15B, RNA binding motif protein 15/15B; ZC3H13, zinc finger CCCH domain-containing protein 13; FTO, fat mass and obesity-associated protein; ALKBH5, AlkB homolog 5; YTHDF1, YTH domain family 1; YTHDC1, YTH domain containing 1; HNRNP, heterogeneous nuclear ribonucleoprotein protein; IGF2BP, insulin-like growth factor 2 mRNA binding protein; eIF3, eukaryotic initiation factor 3.

In the HNRNP family, HNRNPA2/B1 contains two RNA-specific recognition motifs and governs the directional sorting of miRNAs, thereby promoting primary miRNA processing (55). HNRNPC and HNRNPG can modulate mRNA abundance and splicing (43). IGF2BPs have been proven to be a unique and conservative family of m^6^A readers, which can enhance translation efficiency in an m^6^A-dependent way by regulating alternative splicing and improving stability (56). In addition, eIF3 promotes cap (m^7^GPPPN)-independent and YTHDF1-dependent mRNA translation (57). The functions of m^6^A readers are summarized in Table III. The functions of m^6^A regulators are illustrated in Fig. 1.

## 4. m^6^A and BC

Studies have indicated that intricate signal transduction processes at genetic, transcriptomic and epigenetic levels influence the occurrence and progression of cancer, including BC, which is often characterized by genetic and epigenetic alterations (58).

M^6^A methylation has been proven to regulate post-transcriptional gene expression through diverse mechanisms. Different m^6^A readers, writers and erasers interact and crosstalk with each other to activate or inhibit multiple carcinogenic pathways by regulating different targets (59). The abnormal expression of m^6^A mediators in BC is related to different BC subtypes and functions. Changing the degree of m^6^A modification may alter the cell cycle of BC cells and stimulate the proliferation, metastasis and invasion of BC cells by affecting the activity of downstream targets and various signaling pathways, such as the B-cell lymphoma-2 (Bcl-2) and phosphatidylinositol 3-kinase/Protein Kinase B (PI3K/Akt) pathways (60). The disorder of m^6^A regulators is universally existing in BC tissues. The following summarizes the main roles of some important m^6^A regulators in the occurrence and progression of BC.

### METTL3 in BC

As an m^6^A methyltransferase, METTL3 has a crucial role in cancer. Mostly, METTL3 stimulates the occurrence and progression of diverse cancers as an oncogene, through depositing m^6^A modification on key transcripts (37). Numerous studies have validated that METTL3 expression is elevated in diverse cancerous tissues. However, the mechanism by which METTL3 promotes carcinogenesis may differ across various cancer types. The reported mechanisms mainly entail activating multiple m^6^A-dependent signaling pathways, increasing m^6^A modification of carcinogenic primary miR-25 and mediating the binding of m^6^A-modified target transcripts with specific cytokines, so as to promote mRNA translation or degradation, and ultimately facilitate tumor-cell proliferation and migration (61-64).

However, in certain cases, contrary results have been reported for similar tumors, implying that METTL3 may at times function as a tumor suppressor (65). For instance, certain researchers have detected that METTL3 methylation of basic leucine zipper ATF-like transcription factor (BATF) mRNA inhibits its expression in gastric cancer (GC), and low expression of BATF mRNA is significantly associated with postoperative recurrence of GC (66). In addition, there have been reports indicating that the knockdown of METTL3 significantly hastened tumor progression and reduced the lifespan of animals implanted with glioblastoma stem cells (67). Other studies have demonstrated that METTL3 expression is decreased in certain cases of renal cell carcinoma and bladder cancer (68,69). Shi *et al* (70) found that a low level of METTL3 in TNBC is indicative of a poor prognosis, suggesting that the reduced presence of m^6^A markers contributes to the progression of TNBC.

To date, certain studies on the mechanism underlying the role of METTL3 in BC have been published. The present study only provides a summary of recent findings. Wan *et al* (71) discovered that METTL3 enhances the m^6^A modification of programmed cell death ligand 1 (PD-L1) mRNA in BC cells, thereby improving the stability and expression of PD-L1 mRNA. Knocking down METTL3 can boost anti-tumor immunity and reduce PD-L1 expression, thus alleviating the progression of BC. Cai *et al* (72) have shown that the expression levels of METTL3 and hepatitis B x-interacting protein (HBXIP) are very high in BC tissues. HBXIP increases the expression of METTL3 through restraining the expression of tumor suppressor let-7g, and METTL3 in turn upregulates HBXIP via m^6^A modification, thus forming a positive feedback regulatory loop of HBXIP/let-7g/METTL3/HBXIP, and ultimately causing the malignant growth of BC cells (72).

It has also been observed that the METTL3 level in BC is significantly higher than that in surrounding normal tissues, particularly in patients with T3-T4 BC or lymph node metastasis (73). Studies revealed that METTL3 overexpression can upregulate enhancer of zeste homolog 2 through m^6^A modification. This process results in the suppression of tumor suppressor genes and promotion of epithelial-mesenchymal transformation (EMT), which triggers the occurrence, migration and invasion of BC cells (74,75). In addition, another study indicated that METTL3 can accelerate the proliferation of BC by regulating the methylation of BCL-2 or the metastasis associated lung adenocarcinoma transcript 1 (MALAT1)/miR-26b/high mobility group AT-hook 2 axis (76).

**Figure 2:**
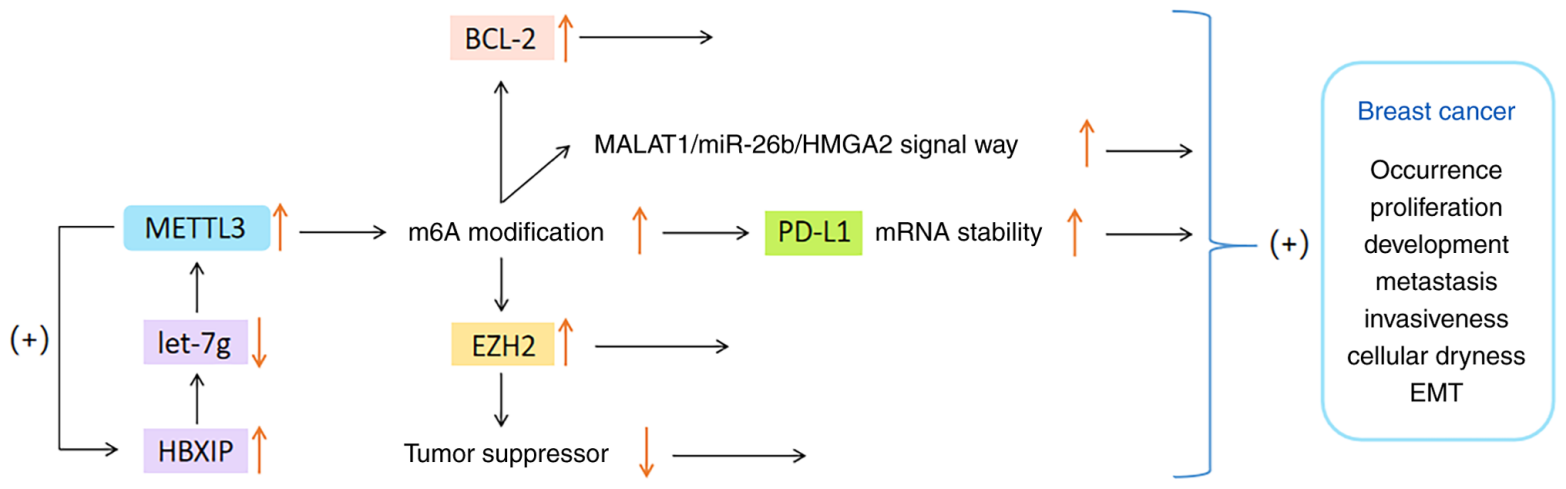
Main functions of METTL3 in breast cancer. METTL3, methyltransferase-like 3; HBXIP, hepatitis B x-interacting protein; let-7g, a kind of tumor suppressor; Bcl-2, B-cell lymphoma-2; EZH2, enhancer of zeste homolog 2; PD-L1, programmed cell death ligand 1; EMT, epithelial-mesenchymal transformation; MALAT1, metastasis associated lung adenocarcinoma transcript 1; miR, microRNA; HMGA2, high mobility group AT-hook 2.

To sum up, METTL3 has been observed to be overexpressed in most BC samples, and its expression level appears to be positively correlated with the malignancy and metastasis of BC. The specific mechanism of the connection between METTL3 and BC-cell proliferation may involve multiple signaling pathways, but the exact mechanism requires to be further studied and clarified. The functions of METTL3 in BC are shown in Fig. 2.

### KIAA1429 in BC

KIAA1429 acts as a scaffold for bridging the core protein of methyltransferase and it is also involved in the positive regulation of diverse tumorigenesis. Certain studies have indicated that KIAA1429 promotes the proliferation and growth of BC in a way independent of m^6^A, and the overall survival period of patients with BC is positively associated with KIAA1429 (77,78).

**Figure 3:**
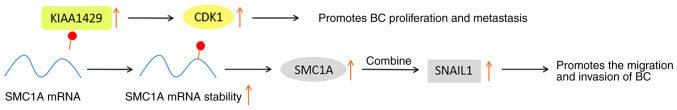
Functions of KIAA1429 in breast cancer. KIAA1429/VIRMA, vir-like m^6^A methyltransferase-associated protein; BC, breast cancer; CDK, cyclin-dependent kinase; SMC1A, structural maintenance of chromosomes 1A; SNAIL, snail family transcriptional repressor.

Zhang *et al* (78) found that KIAA1429 can improve the stability of structural maintenance of chromosomes 1A (SMC1A) mRNA via binding to the motif of SMC1A mRNA. Subsequently, SMC1A further increases snail family transcriptional repressor 1 (SNAIL) expression via binding to the promoter region of the SNAIL gene, which promotes the migration and invasion of BC. Another study illustrated that KIAA1429 targets to regulate cyclin-dependent kinase 1 (CDK1) (77), which is an oncogene related to the proliferation and metastasis of BC. The functions of KIAA1429 in BC are illustrated in Fig. 3.

### FTO in BC

It is known that FTO, as an obesity-related protein, can catalyze the demethylation of m^6^A. Numerous studies have indicated that FTO is significantly upregulated in various cancerous tissues, including but not limited to cervical squamous cell carcinoma (79), lung squamous cell carcinoma (80), gastric cancer (81) and pancreatic cancer (82). FTO is involved in the regulation of tumor progression by decreasing the abundance of m^6^A and activating specific signaling pathways, reducing the overall survival rate of patients afflicted with malignant tumors (83). In a significant proportion of BC specimens, an elevated expression of FTO was observed compared to the adjacent normal breast tissue. Furthermore, it has been strongly associated with tumor proliferation, invasion and metastasis (83-85).

Niu *et al* (84) reported that, in MCF7 and MDA-MB231 cells, the expression of FTO was negatively correlated with BCL2 interacting protein 3 (BNIP3) in BC. Due to the overexpression of FTO in BC, the level of BNIP3 is downregulated, which is necessary for cell apoptosis (84). This change inhibits the cleavage of apoptosis factor caspase-3 and promotes the expression of anti-apoptotic protein Bcl-2 (86,87), thus reducing cell apoptosis and promoting the proliferation and colony formation of BC cells.

Xu *et al* (85) demonstrated that in SKBR3 and MDA-MB453 cells, FTO overexpression decreased the expression of miR-181b-3p, increasing the expression of ADP ribosylation factor like GTPase 5B (ARL5B) directly and indirectly. ARL5B subsequently drives the migration and invasion of HER2+ BC tissue.

Liu *et al* (88) indicated that FTO overexpression promotes aerobic glycolysis and increases ATP production via improving the activity of pyruvate kinase and hexokinase. Subsequently, the PI3K/AKT signaling pathway is abnormally activated, thus accelerating the progression of BC.

**Figure 4:**
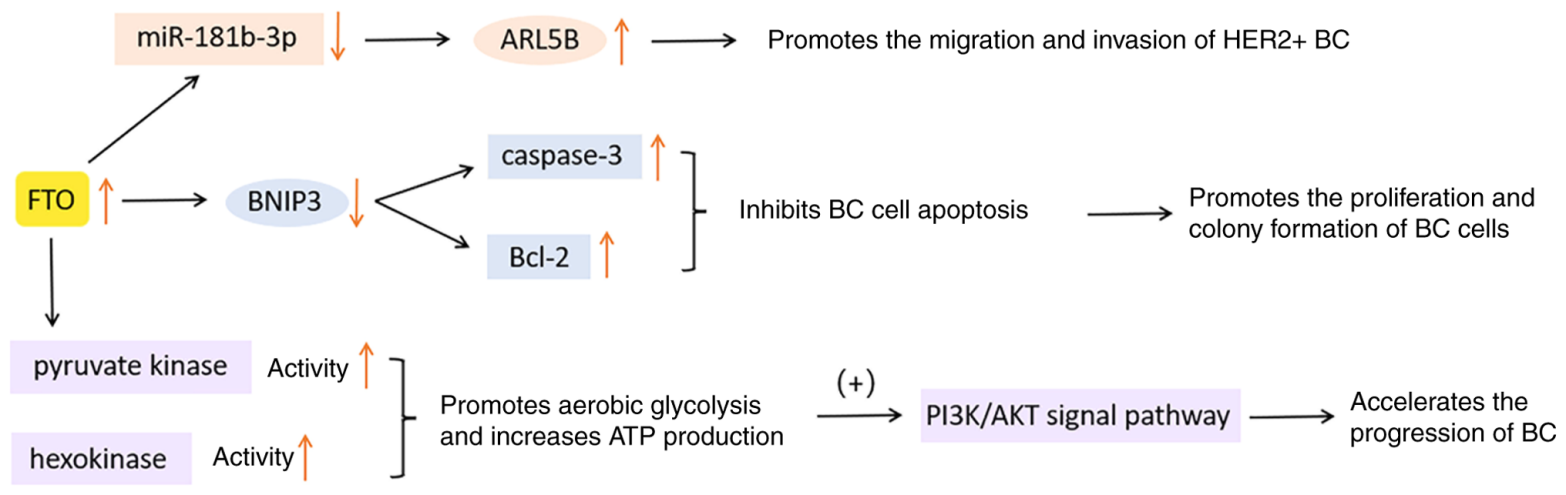
Functions of FTO in breast cancer. FTO, fat mass and obesity-associated protein; miR, microRNA; BC, breast cancer; ARL5B, ADP ribosylation factor like GTPase 5B; Bcl-2, B-cell lymphoma-2; BNIP3, BCL2 interacting protein 3; PI3K, phosphatidylinositol 3-kinase; AKT/PKB, protein kinase B.

In conclusion, deregulation of FTO is a tumorigenic factor that cannot be ignored. The FTO-m^6^A axis can be considered a potential new target for the treatment and diagnosis of BC. The functions of FTO in BC are presented in Fig. 4.

### ALKBH5 in BC

A growing body of evidence indicates that ALKBH5 is commonly dysregulated in malignant tumors, which regulates the expression of multiple oncogenes and contributes to tumor immune evasion through post-transcriptional mechanisms (89). However, studies indicated that ALKBH5 has a dual role in cancer, as its expression is not consistently upregulated or downregulated across all cancer types. Certain studies have shown a positive association between ALKBH5 levels and BC (90-92).

Under anoxic conditions, ALKBH5 mediates the pluripotency factor Nanog homeobox (NANOG) to regulate the BC stem cell characteristic specification in a hypoxia-inducible factor-dependent manner. In other words, ALKBH5 enhances the demethylation of NANOG mRNA and upregulates NANOG, while knocking down ALKBH5 inhibits this pluripotency factor (93,94). Therefore, ALKBH5 disorder is considered to be an important link in the proliferation, metastasis and enhancement of the stem cell phenotype of BC.

In addition, ALKBH5 upregulates the expression of ubiquitin conjugating enzyme E2 C (UBE2C) and reduces that of p53 by modifying the m^6^A of the downstream target gene UBE2C (91). Among them, UBE2C has been proven to exert a carcinogenic effect (95). The upregulated p53 is conducive to decreasing cancer cells and preventing the occurrence of cancer (96). Therefore, the ALKBH5/UBE2C/p53 axis is regarded as a potential mechanism for promoting the tumorigenesis and metastasis of TNBC cells (91).

**Figure 5:**
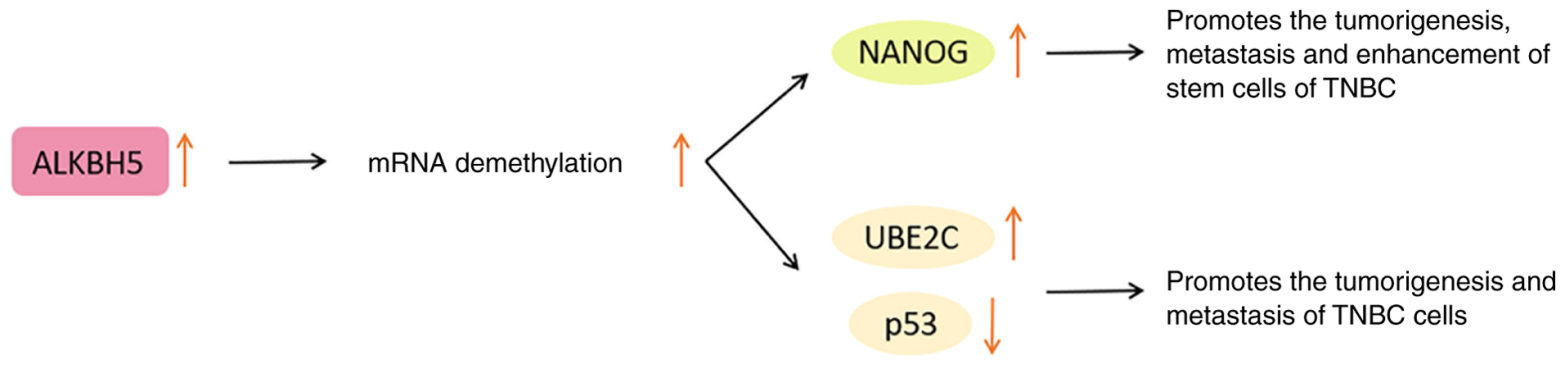
Functions of ALKBH5 in breast cancer. ALKBH5, AlkB homolog 5; NANOG, Nanog homeobox; UBE2C, ubiquitin conjugating enzyme E2 C; p53, tumor protein 53; TNBC, triple-negative breast cancer.

In general, before ALKBH5 can be utilized as a therapeutic target for BC, its expression and specific regulatory mechanism should be further clarified. The functions of ALKBH5 in BC are presented in Fig. 5.

### YTHDFs in BC

As a m^6^A binding protein, YTHDF1 amplification is universal in cancer tissues. The level of YTHDF1 is negatively associated with survival and positively correlated with the degree of malignancy and metastasis (97-99). In the experimental report by Sun *et al* (98), YTHDF1 and its downstream target transcription factor 8 (E2F8) were indicated to promote the transition to S-phase by regulating cell cycle-related factors, and to be involved in DNA replication and DNA damage repair (DDR). Furthermore, YTHDF1 blocked the cleavage of E2F8 mRNA, which is dependent on METTL14. All of these findings indicate that YTHDF1 functions as a promoter of tumor growth. As reported by Chen *et al* (97), low YTHDF1 restrained the proliferation, invasion and EMT of BC cells, and blocked cell-cycle progression. YTHDF1 also accelerated the translation of forkhead box (FOX)M1 by combining with m^6^A-modified FOXM1 mRNA, thus promoting its carcinogenic effect.

YTHDF2 can selectively bind m^6^A-modified sites and promote mRNA decay, but its function in solid tumors is still controversial. Recent reports have mentioned that YTHDF2 can degrade tumor promoter and tumor suppressor gene mRNA and have a dual role in tumor progression (100,101). For instance, YTHDF2 acts as a cancer-promoting regulator in certain tumors, such as glioblastoma, acute myeloid leukemia and prostate cancer (102-104). However, it has a tumor suppressor function in other tumor types, such as melanoma and liver cancer (100). As Einstein *et al* (105) suggested, moderate expression of YTHDF2 is essential to maintain the survival of cells driven by MYC proto-oncogene, bHLH transcription factor (MYC). Depletion of YTHDF2 activates the EMT-specific pathway in BC cells, particularly in TNBC, leading to further activation of cancer-related translation initiation factors. However, in MYC-addicted cells, over-translation of these target mRNAs eventually activates programmed cell death, leading to TNBC tumor-cell apoptosis. This result proves the importance of YTHDF2 for the survival of TNBC cells and the feasibility of knocking down YTHDF2 as a potential therapeutic method.

YTHDF3 may boost translation by interacting with ribosomal protein and significantly raise the translation efficiency of YTHDF1/3 common target (106). YTHDF3 can enhance the stability of its target factor zinc finger E-box binding homeobox 1 (ZEB1) mRNA, which is an EMT transcription factor (107). Chang *et al* (108)'s study on brain metastasis of BC indicated that YTHDF3 regulates its own mRNA translation by binding to m^6^A residues in its 5'UTR. YTHDF3 also combines with m^6^A-modified mRNA to promote the expression of brain metastasis genes, such as ST6 N-acetylgalactosaminide α-2,6-sialyltransferase 5, gap junction protein α1 and EGFR. It is noteworthy that in comparison to primary BC, YTHDF3 expression was significantly increased in its brain metastases, but not in other organs such as lung, bone, liver, spleen, lymph nodes and adrenal glands.

**Figure 6:**
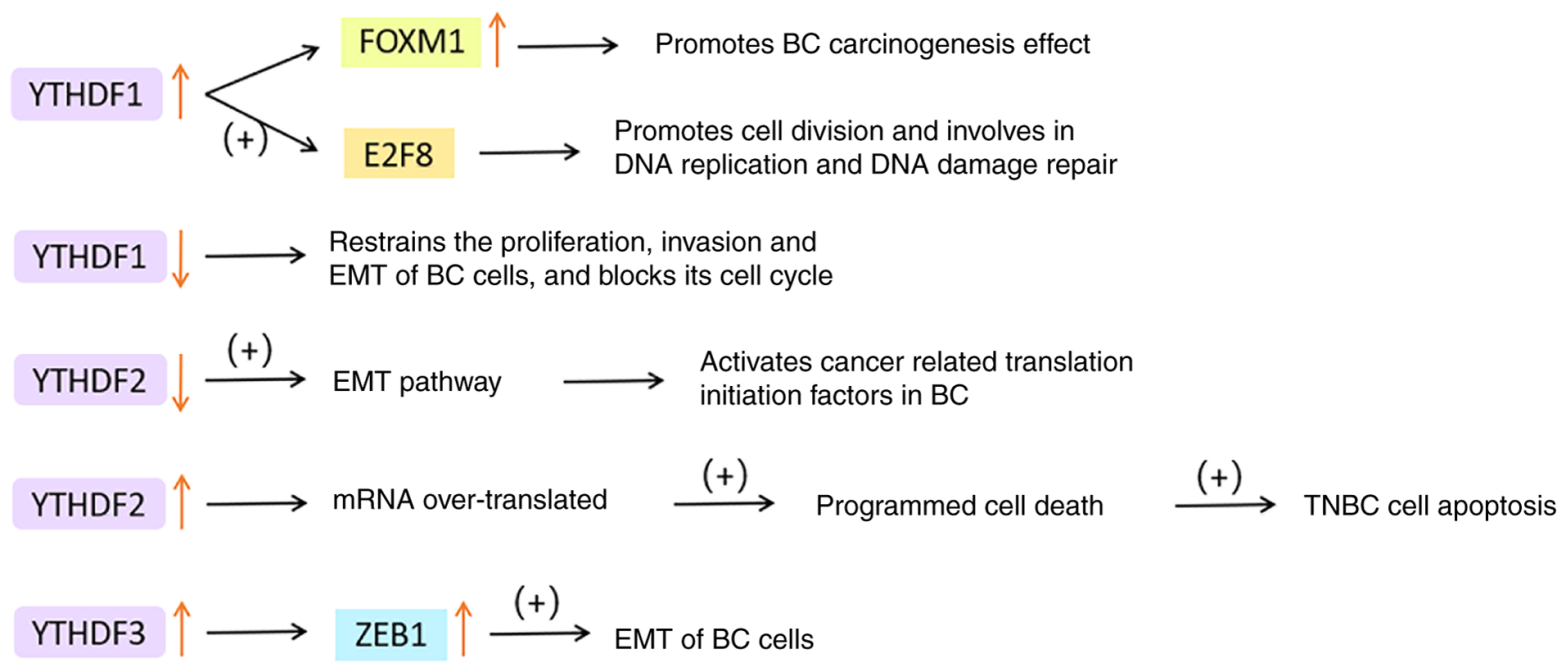
Functions of YTHDFs in breast cancer. YTHDF1, YTH domain family 1; FOXM1, forkhead box M1; E2F8, transcription factor 8; TNBC, triple-negative breast cancer; EMT, epithelial-mesenchymal transformation; ZEB1, zinc finger E-box binding homeobox 1.

In conclusion, YTHDF disorder is a prevalent occurrence in cancer tissues. YTHDF1 and YTHDF3 are responsible for improving the translation efficiency of m^6^A-modified mRNA, and they are frequently amplified in BC cells. Their high levels are closely related to poor prognosis and low survival rates. Conversely, YTHDF2 promotes mRNA degradation and also acts as a carcinogen most of the time. It is plausible that the YTH family proteins work collaboratively to execute their regulatory role in translation, but their respective roles in cancer cannot be replaced, providing potential targets for BC treatment. The functions of YTHDFs in BC are displayed in Fig. 6.

### IGF2BPs in BC

IGF2BP protein is a newly discovered m^6^A binding protein, which selectively binds to mRNA transcripts (109). The carcinogenic effect of IGF2BPs depends on its function of improving the stability and translation efficiency of certain oncogene mRNAs, such as MYC (109,110). The translocation of IGF2BPs may lead to the anomalous accumulation of carcinogenic products, thus stimulating the malignant development of cancer tissue (109).

Qiao *et al* (111) found that long intergenic ncRNA 483 (LINC00483) can promote the proliferation of BC cells and is negatively associated with the survival rate of patients with BC. A high level of IGF2BP1 significantly increased the expression of LINC00483, thus inducing carcinogenesis. Shi *et al* (112) reported that proto-oncogene MYCN activates IGF2BP1, and subsequently, IGF2BP1 enhances the stability of the carcinogen miR210HG and mediates its carcinogenic function in BC. According to the latest research, the ubiquitin specific peptidase 10 (USP10)/IGF2BP1/carnitine palmitoyl transfer 1A (CPT1A) axis plays an important role in BC metastasis (113). They found that the de-ubiquitination enzyme USP10 reduces its cleavage by de-ubiquitination of IGF2BP1. Subsequently, IGF2BP1 binds to the m^6^A site on CPT1A mRNA and makes it more stable, thus promoting the growth and metastasis of BC (114,115).

In BC, the level of PD-L1 increases with the increase of IGF2BP3. Knocking down IGF2BP3 significantly inhibited the expression of PD-L1, which cooperates with tumor cells to escape immune surveillance (70). In addition, the IGF2BP3/tripartite motif containing 25 (TRIM25)/miR-3614 axis represents a new way to regulate tumor cell proliferation. TRIM25 is mainly expressed in estrogen target tissues, which can improve cell viability and promote cell proliferation. MiR-3614-3p can be used as a tumor suppressor to inhibit the growth of BC cells. IGF2BP3 can induce the expression of TRIM25 and inhibit the maturation of miR-3614, which conversely protects TRIM25 mRNA from miR-3614-mediated degradation (116).

**Figure 7:**
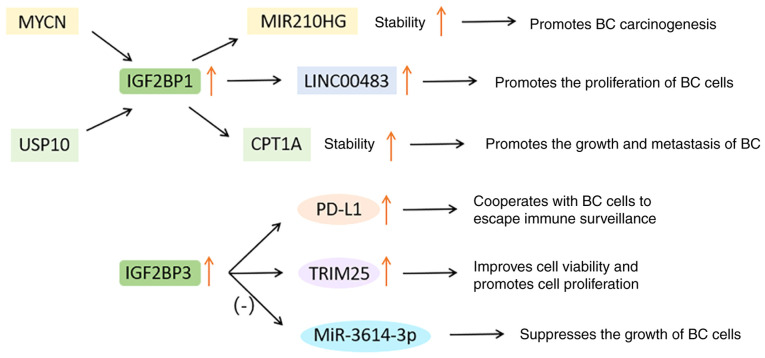
Functions of IGF2BPs in breast cancer. IGF2BP, insulin-like growth factor 2 mRNA binding protein; MYCN, a kind of proto-oncogene; USP10, ubiquitin specific peptidase 10; MIR, microRNA; LINC00483, long intergenic ncRNA 483; CPT1A, carnitine palmitoyl transfer 1A; PD-L1, programmed cell death ligand 1; TRIM25, tripartite motif containing 25.

In short, the IGF2BP gene and its downstream targets are generally amplified in BC, thereby resulting in enhanced proliferation, metastasis and poor prognosis. These results provide a foundation for evaluating IGF2BP as a potential target for BC treatment, while the specific mechanism of IGF2BP should be further studied. The functions of IGF2BPs in BC are shown in Fig. 7.

### Other m^6^A regulators in BC

The dysfunction of METTL14, WTAP, RBM15/15B and ZC3H13 in methyltransferase are also commonly recorded in cancer databases. METTL14 has been reported as an oncogene in most studies and its expression is usually positively correlated with the expression of METTL3 and WTAP. It can improve the stability of target mRNA through HuR (RNA-binding protein) mediation, involving in the regulation of cell cycle, EMT and other tumor growth processes (90). The expression level of WTAP in BC is higher than that in normal breast tissue, and it is positively correlated with tumor size and grade (117). Certain scholars have reported that the complement C5a receptor 1 (C5AR1)+/WTAP/enolase 1 (ENO1) axis regulates the glycolytic activity of BC cells and the lncRNA DLG-associated protein 1-antisense 1/miR-299-3p/WTAP axis promotes the proliferation of drug-resistant BC cells, which is worthy of further exploration (118,119). However, the function of WTAP in tumors cannot be separated from the expression of METTL3 (120).

**Figure 8:**
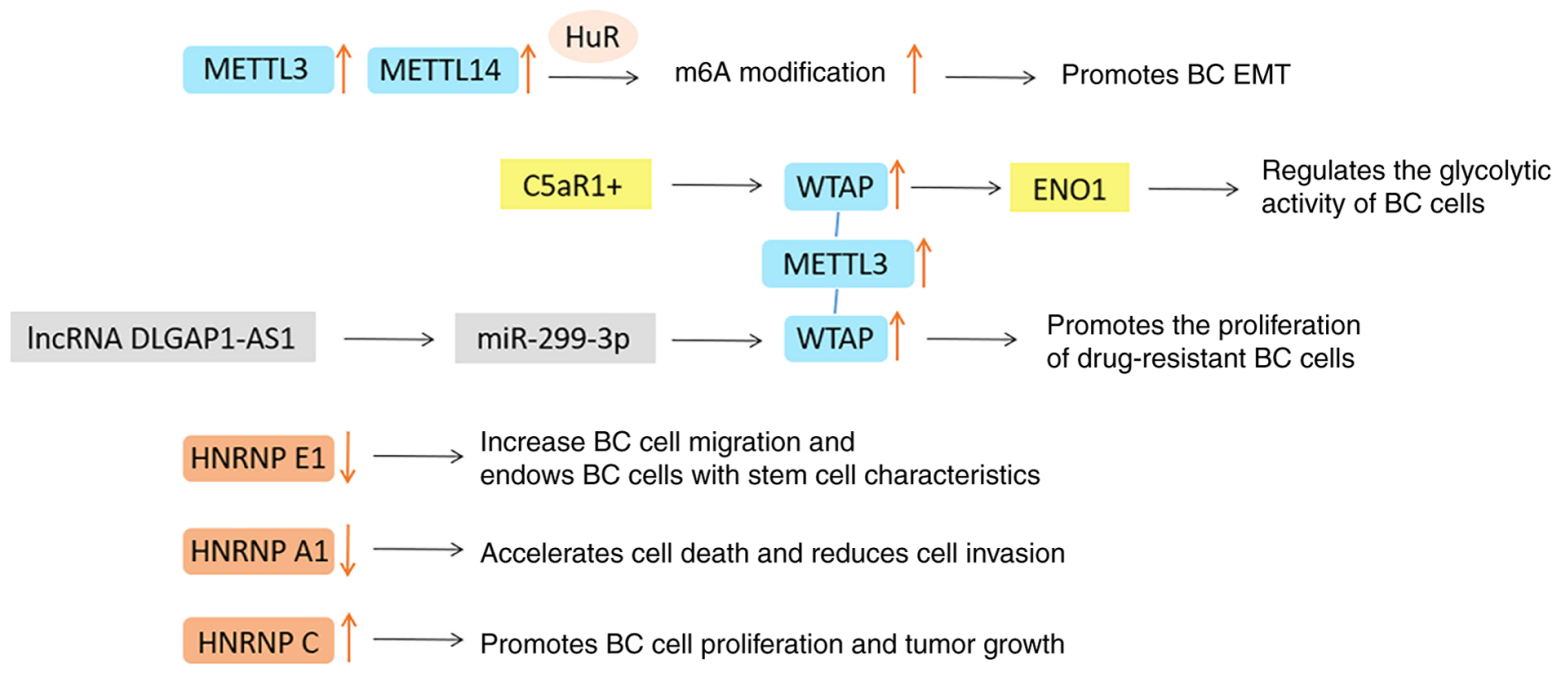
Functions of other m6A regulators in breast cancer. m^6^A, N^6^-methyladenosine; METTL3, methyltransferase-like 3; HuR, a kind of RNA-binding protein; BC, breast cancer; EMT, epithelial-mesenchymal transformation; C5aR1, C5a receptor 1; WTAP, Wilms tumor 1-associated protein; ENO1, enolase 1; miR, microRNA; HNRNP, heterogeneous nuclear ribonucleoprotein protein.

HNRNPs, another family of m^6^A readers, are also involved in regulating various types of RNA processing, including translation and splicing (121). The tumor suppressor HNRNP E1 regulates the expression of EMT-related genes. Silencing HNRNP E1 increased BC-cell migration and endowed cells with stem cell characteristics, which promoted abnormal proliferation and metastatic growth of cancer cells (122). Knocking down HNRNP A1 accelerated cell death and reduced cell invasion (123). HNRNPC is upregulated in diverse cancers and HNRNPC silencing significantly suppressed BC-cell proliferation and tumor growth (124). The functions of these regulators in BC are illustrated in Fig. 8.

## 5. M6A modification and genomic instability in BC

Genomic instability is a hallmark of cancer and refers to the increased rate at which cells acquire genomic alterations (125). Certain regulatory factor-mediated m^6^A modifications have been linked to genomic instability, specifically in terms of regulating the effect of m^6^A modification on DNA damage and repair processes (126). While this relationship has been established and verified in numerous studies pertaining to tumors, investigations into its role in BC remain scarce.

### M6A modification and genomic instability

METTL3 can be specifically recruited to gene fragments damaged by ultraviolet radiation and rapidly methylated RNA; subsequently, m^6^A-modified RNA starts the DDR pathway to improve the cell survival rate (126,127). METTL3-mediated m^6^A methylation also regulates homologous recombination (HR)-mediated double-stranded DNA break (DSB) repair (128). Phosphorylated METTL3 can be localized in the DSB region so that the damaged chromatin region of the RNA is modified by m^6^A. The m^6^A-modified RNA is then recognized by YTHDC1 and forms a DNA-RNA hybrid with DSBs, which recruits repair-related proteins and promotes HR-mediated repair (129). It has been reported that a low level of METTL3 increases the sensitivity of cancer cells to the treatment of DNA damage, while upregulated METTL3 reduces the survival rate of patients with head and neck squamous cell carcinoma who have received cisplatin or radiation treatment of DNA damage (126). Knocking down METTL3-mediated and YTHDC2-mediated m^6^A modification led to the accumulation of DNA-RNA hybridization (R loop) and γH2AX (a DSB marker), which plays a key role in inhibiting cell growth and regulating genome stability (130).

A study revealed that METTL3-mediated m^6^A modification improves the stability of transcription factor activated enhancer binding protein 2C mRNA, thus increasing the abundance of DNA repair genes, which endows spermatogonioma cells with resistance to DNA damage induced by cisplatin treatment, promoting tumor cell survival (131). METTL14 arginine methylation is positively correlated with enhanced translation of DNA repair genes (132). VIRMA was also demonstrated to enhance the invasion and cisplatin resistance of teratoma cells by regulating DNA damage (133). On the contrary, FTO participates in the upregulation of repair gene resection repair cross complementation group 1 through β-catenin mRNA demethylation, equipping cervical squamous cell carcinoma with radiochemotherapy resistance (79,134). ALKBH5 can also be inhibited by small ubiquitin-like modifier, which upregulates DNA repair genes and protects cells from reactive oxygen species (ROS)-induced DNA damage (126). This indicates that m^6^A plays a dual role in anti-cancer therapy based on DNA damage through DDR.

M^6^A modification is also involved in the regulation of telomere length and genomic integrity in human cancer (135). Telomere shortening is closely associated with cancer-related genomic changes (136). Homebox-containing protein 1 (HMBOX1) is a telomere-binding protein. HMBOX1 mRNA has been identified as the real target of m^6^A modification in cancer cells. HMBOX1 degradation caused by upregulation of METTL3 and YTHDF2 in cancer cells leads to telomere shortening and dysfunction of p53-dependent DNA damage response pathway inactivation. This change is likely to lead to various types of telomere-related chromosome aberrations, thus enhancing the tumorigenicity and invasiveness of cancer cells. Conversely, the malignant progression of cancer cells caused by METTL3-induced genomic instability can be alleviated or even reversed by introducing HMBOX1 (135).

### M^6^A modification and genomic instability in BC

The pathogenesis of BC primarily entails the hyperactivation and overexpression of oncogenes, coupled with deficiencies in DDR gene defects, DDR gene transcription defects and mitotic defects, among others. The defective repair of damaged DNA leads to genomic instability, which is closely related to the malignant progress and poor prognosis of BC.

It has been found that tumor genome subtypes of BC are related to tumor gene expression, which involves the methylation gain and loss processes of a large number of loci (137). The researchers suggested that extensive aberrations in methylation induce epigenomic instability, rendering tumors more prone to regulatory mutation and deterioration. They even linked different methylation scores with higher epigenetic instability and higher chromosomal instability in BC, predicting the disease stage and progress (137).

Based on the above principles and experimental evidence, it may be reasoned that METTL3-mediated modification of m^6^DSB repair may serve as a promising target for cancer treatment, including BC. Whether targeted inhibition of METTL3 can reduce the proliferation activity and invasiveness of BC cells by inhibiting DNA repair or improve the sensitivity of BC cells to DNA damage therapies (such as chemotherapy or radiotherapy) is also likely to become a new topic. It may also be true for other m^6^A methylases and demethylases.

## 6. M^6^A modification and therapeutic resistance in BC

One of the main reasons for reduced efficacy of non-surgical treatment for tumors is drug resistance of tumor cells. Intrinsic resistance is mainly related to gene mutations, while acquired resistance refers to a weakened response to drugs after treatment, which may be related to secondary mutations in drug targets (138). In recent years, research on the role of m^6^A regulators in drug resistance in cancer treatment has made significant progress, which has also been confirmed in the treatment of BC (139).

Tamoxifen chemotherapy, as a first-line endocrine therapy option for BC, is facing a major problem of drug resistance. Research has proved that long-term exposure to tamoxifen can induce an increase in METTL3 expression, further resulting in an increase in m^6^A of the 5'UTR of adenylate kinase 4 (AK4; a mitochondrial nucleotide kinase) mRNA. High levels of AK4 inhibit mitochondrial apoptosis and promote ROS production, activating p38, ultimately leading to increased resistance of MCF-7 cells to tamoxifen (140). High-expression HNRNPA2B1 in endocrine-resistant MCF-7 and LCC9 BC cell lines endows cancer cells with acquired endocrine resistance by activating the Ser/Thr kinase growth factor signaling pathway that regulates its downstream target (141). The increased expression of activating transcription factor 3 (ATF3) protein caused by low levels of YTHDF2 is also the reason for the development of tamoxifen-resistant MCF-7 cells (142). Therefore, selective inhibition of AK4, HNRNPA2B1 and ATF3 may serve as a potential strategy for preventing BC cells from acquiring endocrine therapy resistance.

Similarly, the abnormal expression of m^6^A regulatory factors can make BC cells resistant to certain chemotherapy drugs. Anthracyclines have been considered to be the most effective chemotherapeutic drugs for BC in recent years, but are facing serious drug resistance problems. Research has shown that miR-221-3p is an miRNA involved in tumor development, metastasis and drug resistance. High levels of METTL3 increase the expression of miR221-3p and negatively regulate homeodomain interacting protein kinase 2, a tumor suppressor that can be activated by doxorubicin, thereby reducing the efficacy of doxorubicin (143). The latest research shows that METTL3 and YTHDC1 promote the synthesis of EGF and DNA repair protein RAD51 recombinase (RAD51) and improve HR and cell survival during doxorubicin treatment, resulting in drug resistance of BC cells by co-regulating m6A-modified related mRNA (144). Li *et al* (145) found that the m^6^A modification of METTL3 increased the level of MALAT1 protein, recruited E2F1 and activated the transcription of downstream anterior gradient 2, protein disulphide isomerase family member, contributing to doxorubicin resistance in BC. Wu *et al* (146) found that ALKBH5 removes m^6^A modification to stabilize BRCA1 (DNA repair protein) mRNA, further enhance its DNA repair ability and increase the resistance of BC cells to doxorubicin. Wang *et al* (147) have shown that FTO mediates doxorubicin resistance in BC by activating signal converters such as transcription activator STAT3 in BC. ALKBH5-mediated FOXO1 m^6^A demethylation increases the expression of superoxide dismutase 2 and leads to a lower ROS level, thus promoting the maintenance of cancer stem cell characteristics and doxorubicin resistance in TNBC. Furthermore, a study indicated that targeted inhibition of FOXO1 both *in vivo* and *in vitro* can restore the drug sensitivity of TNBC (148). The exosomal Piwi-interacting RNA-17560 derived from senescent neutrophils enhances the stability and expression of ZEB1 transcript by upregulating FTO levels, leading to chemical resistance and EMT in tumor cells (149).

Radiation resistance refers to the adaptability of tumor cells or tissues to radiation therapy. DDR is one of the main reasons for tumor cells to develop radiation resistance (150). The transmembrane glycoprotein neuropilin 1 (NRP1) can enhance the stem cell characteristics of BC cells, making them resistant to radiation therapy. NRP1 has been shown to reduce cell apoptosis and enhance radiation resistance by downregulating Bcl-2 through m^6^A methyltransferase WTAP (151).

## 7. Summary and outlook

So far, the role of m^6^A methylation in cancer remains in the preliminary stages of research and its application in clinical targeted therapy is limited. The available studies consistently show that m^6^A is subject to dynamic reversible modification by three distinct types of regulators, which can effectively regulate mRNA splicing, translation, stability and decay. Through specific mRNA modification, m^6^A can regulate the expression of target genes and consequently impact tumor progression. However, these target genes contain oncogenes and tumor suppressor genes, and the change trend of m^6^A regulatory factors differs from one type of cancer tissue to another, which means that m^6^A plays a dual role in tumor progression.

Furthermore, the interaction between m^6^A methylation and tumor metabolism is complex. Tumor metabolic stress can abnormally regulate m^6^A methylation, while disorder of m^6^A methylation can in turn regulate signaling pathways related to tumor metabolism. Other studies have found that m^6^A is involved in regulating the metabolic reprogramming of BC (7). For instance, C5AR1-positive neutrophils are capable of secreting IL-1β and TNF-α, which activate ERK1/2 signaling to enhance WTAP stability. The upregulated WTAP subsequently elevates the expression of ENO1, thereby promoting glycolysis and ultimately facilitating the progression of BC (118). In addition, breast tumor cells develop resistance to radiation, chemotherapy and endocrine therapy drugs due to the abnormal expression of certain m^6^A regulatory factors and regulation of specific signaling pathways. Therefore, targeting upregulation or downregulation of certain m^6^A-related genes and activating or inhibiting certain m^6^A regulatory factors can enhance the sensitivity of tumors to radiotherapy, chemotherapy and endocrine therapy (150). It provides an innovative idea for the combination treatment of BC and the development of new drugs.

At present, joint research on m^6^A and BC focus on investigating the correlation and specific regulatory mechanism linking the expression of certain m^6^A regulatory factors with their corresponding target genes, as well as with various BC subtypes, malignancy, metabolism, growth, metastasis, immunity, drug resistance and adverse prognosis. While the experimental findings hold some informative value, their reliability and applicability necessitate further validation through larger BC sample sizes and clinical implementation. In conclusion, research regarding m^6^A and BC holds promise for yielding diagnostic and therapeutic breakthroughs in the treatment of BC. However, these models and methods necessitate further refinement and the mechanism by which m^6^A-related proteins regulate the progression of BC is required to be further explored.

The majority of the experimental findings indicate that m^6^A regulators are imbalanced in BC. Based on the function of m^6^A modification in tumor tissue and the close correlation between m^6^A regulatory factors and tumors, many scholars claim that targeted m^6^A methylchemotherapy is likely to become a promising option for tumor treatment. Consequently, exploring the optimal strategy and conducting clinical trials on the combination of m^6^A enzyme-related drugs and m^6^A targeted therapy will likely represent a novel direction for BC treatment in the future. These strategies require the determination of the specific pathways and mechanisms through which m^6^A impacts the progression of BC, serving as the theoretical foundation. This paper summarizes the relevant mechanisms that have been found so far, yet it should be acknowledged that numerous challenges remain to be overcome.

First, m^6^A regulates the progression of BC by complex mechanisms, involving a variety of regulatory factors, signaling pathways and oncogenes. Modifying any of these pathways may trigger a series of associated reactions, which should be predicted before implementing targeted therapy. Furthermore, certain m^6^A regulators play dual roles in BC, so it is necessary to consider tumor heterogeneity and specific maladjustment factors before designing a treatment plan.

In addition, a variety of serious problems should be considered when screening potential targeted drugs. For instance, the specific ways that m^6^A-related drugs affect the methylation level, whether these drugs have cytotoxicity, whether they are generally applicable to different subtypes of BC and how to deal with BC resistance should be determined. In addition, it is worth noting that different subtypes of BC exhibit varying degrees of different sensitivity to radiotherapy, chemotherapy, immunotherapy and various drugs, and they are regulated by m^6^A modification, which greatly affects the treatment effect.

Finally, in-depth exploration of cancer epigenomics and the advancement of high-quality nucleic acid probes facilitate the precise identification of biomarkers, which is essential for predicting potential therapeutic targets, individualized treatment and improvement of prognosis. By resolving these challenges, the prospect of m^6^A targeted therapy for BC will expand significantly.

## Figures and Tables

**Table I:** Functions of m^6^A 'writers'.

Regulator	Effect on m^6^A modification	(Refs.)
METTL3	Catalytic m^6^A modification; core protein of methyltransferase	(31,36)
METTL14	Catalytic m^6^A modification; allosteric activator of METTL3	(37)
WTAP	Recruits METTL3-METTL14 heterodimer into the nuclear speckles	(38)
METTL16	Catalyzes m^6^A modification; deposits N^6^ into particular mRNA targets	(39)
VIRMA (KIAA1429)	Mediates preferential m^6^A deposition in the 3'UTR	(40)
RBM15/15B	Recruits methyltransferase complex to specific RNA sites	(41)
ZC3H13	Anchors WTAP in the nucleus and promotes m^6^A methylation	(42)

m^6^A, N^6^-methyladenosine; METTL3, methyltransferase-like 3; WTAP, Wilms tumor 1-associated protein; VIRMA, vir-like m^6^A methyltransferase-associated protein; RBM15/15B, RNA binding motif protein 15/15B; ZC3H13, zinc finger CCCH domain-containing protein 13.

**Table II:** Functions of m^6^A 'erasers'.

Regulator	Effect on m^6^A modification	(Refs.)
FTO	Removes m^6^A modification; regulates pre-nuclear mRNA processing	(44)
ALKBH5	Removes m^6^A modification; mediates the transport, metabolism and assembly of mRNA	(43)

m^6^A, N^6^-methyladenosine; FTO, fat mass and obesity-associated protein; ALKBH5, AlkB homolog 5.

**Table III:** Functions of m^6^A 'readers'.

Regulator	Effect on m^6^A modification	(Refs.)
YTHDF1	Promotes the translation of m^6^A-modified RNA	(48)
YTHDF2	Promotes the degradation of m^6^A-modified RNA	(49,50)
YTHDF3	Promotes the translation and degradation of m6A-modified RNA	(51)
YTHDC1	Promotes RNA splicing and export	(47,53)
YTHDC2	Improves the translation efficiency of target mRNA	(54)
HNRNPA2/B1	Promotes primary microRNA processing	(55)
HNRNPC and HNRNPG	Promotes mRNA abundance and splicing	(43)
IGF2BPs	Promotes the stability, splicing and translation of mRNA	(56)
eIF3	Promotes mRNA translation	(57)

m^6^A, N^6^-methyladenosine; YTHDF1, YTH domain family 1; YTHDC1, YTH domain containing1; HNRNP, heterogeneous nuclear ribonucleoprotein protein; IGF2BP, insulin-like growth factor 2 mRNA binding protein; eIF3, eukaryotic initiation factor 3.

## Data Availability

Not applicable.
